# Collapse Mechanism of Single-Layer Cylindrical Latticed Shell under Severe Earthquake

**DOI:** 10.3390/ma13112519

**Published:** 2020-06-01

**Authors:** Haitao Zhou, Yigang Zhang, Feng Fu, Jinzhi Wu

**Affiliations:** 1 School of Civil and Transportation, Henan University of Urban Construction, Pingdingshan 467001, China; zhouhaitao@hncj.edu.cn; 2 Spatial Structure Research Center, Beijing University of Technology, Beijing 100124, China; bzy@bjut.edu.cn (Y.Z.); kongjian@bjut.edu.cn (J.W.); 3 Key Lab of Urban Security and disaster Engineering, Ministry of Education, Beijing University of Technology, Beijing 100124, China; 4 School of Mathematics, Computer Science & Engineering, Northampton Square, City, University of London, London EC1V0HB, UK

**Keywords:** cylindrical latticed shell, damage accumulation, progressive collapse, finite element, earthquake

## Abstract

In this paper, the results of finite element analyses of a single-layer cylindrical latticed shell under severe earthquake are presented. A 3D Finite Element model using fiber beam elements is used to investigate the collapse mechanism of this type of shell. The failure criteria of structural members are simulated based on the theory of damage accumulation. Severe earthquakes with peak ground acceleration (PGA) values of 0.5 g are applied to the shell. The stress and deformation of the shell are studied in detail. A three-stage collapse mechanism “double-diagonal -members-failure-belt” of this type of structure is discovered. Based on the analysis results, measures to mitigate the collapse of this type of structure are recommended.

## 1. Introduction 

After the events of 11 September 2001, more and more researchers have carried out research work on the investigation of the mechanism of progressive collapse in buildings, trying to find possible mitigating methods. However, few studies have been performed for space structures. As a typical long-span space structure, single-layer cylindrical reticulated domes are widely used in public buildings, such as terminals, gymnasiums, large factories and so on due to their graceful appearance. However, if a dome collapses due to a severe earthquake, heavy casualties and economic losses would be caused. Therefore, research in this area is imperative. 

In the current design practice, some design procedures are available in Europe and the United States on mitigating the progressive collapse of buildings, such as the design guidance of the Department of the American Concrete Institute (ACI) [1] and the General Services Administration (GSA) [2] in which specific processes of assessment on the necessity of anti-collapse design are recommended. According to the American Society of Civil Engineers (ASCE) [3] ductility and the sufficient connection performance of the structure are required. In Eurocode 1 [4], it is stipulated that the structure must be able to resist a certain accidental load without a large-scale collapse. Those specifications aimed at mitigating the collapse of structures due to specific accidents. In Eurocode 2 [5], special requirements are given to the construction of reinforced concrete, especially about the anchorage and connection of bars. Some references, such as FEMA [6] and NIST [7], also provide general design recommendations, which require steel-framed structural systems to have enough redundancy and resilience. However, as most of these design codes focused on buildings, no detailed design procedures to prevent the progressive collapse of reticulated shells are available.

To date, some numerical investigations and experimental tests have been carried out on building structures. In 1974, McGuire [8] reviewed the problem of progressive collapse of multi-story masonry buildings under abnormal loading and presented some recommendations to prevent progressive collapse. Shimada [9] conducted a shaking table test of a full-scale two-story and 1 × 1 span steel frame model in 2007. Song [10] conducted a test on a steel frame building by the sudden physical removal of four columns at ground level to investigate the load redistribution within the building. Chen et al. [11] conducted a progressive collapse resistance experiment on a two-story steel frame in which a composite concrete slab was adopted. Tsitos [12] performed quasi-static “push-down” experiments on a 1:3 scale three-story steel frame, considering multi-hazard extreme loading. Starossek [13] suggested a pragmatic approach for designing against progressive collapse and a set of design criteria. Lim [14] investigated the progressive collapse of 2D steel-framed structures with different connections and found that horizontal column buckling propagation control was the only solution. Yamazaki [15] clarified the frame conditions that enable stories to resist progressive collapse through comparing the gravity potential energy released by the story collapse with the energy columns absorb before they completely collapse due to the compressive load. O’Dwyer [16] presented details of an algorithm for modeling the progressive collapse of framed multi-story buildings. Xingxing Chen et al. [17] presents an experiment and analysis on a 1/5-scaled 10-story SRC frame- structural model. Fu [18,19] made numerical simulation of a multi-story building under consecutive column removal scenarios and Gao et al. [20] performed experimental investigation on semi-rigid composite joints to accommodate column loss. Qian et al. [21] investigated the behavior of precast concrete frames with high-performance dry connections subjected to the loss of a penultimate column.

However, most of the above research is related to building structures, while little has been carried out on long-span space structures. Fu et al. [22] performed a numerical simulation for the progressive resistance of a double-layer grid space structure. Zheng et al. [23] developed a force displacement hysteresis model for the collapse simulation of a power transmission pylon, considering the buckling/softening-based fracture criterion. Rashidyan et al. [24] recommended that method of strengthening the compression layer members along with weakening the tension layer members of double-layer space trusses was an effective method for increasing the structure’s ductility and load-bearing capacity against progressive collapse. To study the progressive collapse phenomenon of structures during earthquakes, Lau et al. [25] carried out nonlinear analyses of reinforced concrete bridges by the Applied Element Method (AEM). Miyachi et al. [26] carried out a progressive collapse analysis for three continuous steel truss bridge models with a total length of 230.0 m using large deformation and elastic plastic analysis. Takeuchi et al. [27] proposed post-fracture analysis methods for truss structures composed of tubular members with large diameter-to-thickness ratios, and investigated the collapse mechanism of such truss towers after the buckling and fracture of the main columns and diagonals. Ponter et al. [28] discussed the programming method based on the Elastic Compensation method used for limit and shakedown analyses of steel structures. Skordeli et al. [29] studied examples of limit and push-down analyses of spatial frames under the aforementioned ellipsoidal approximations, with several aspects discussed, starting from a computational equation for the elastoplastic limit analysis of 3D truss–frame systems, apt to provide the exact limit load multiplier and attached collapse mechanism. Ferrari et al. [30] derived a full evolutive piecewise linear response from a bridge for different loading configurations. Xia et al. [31] studied the dynamic behavior of snap-through buckling in single-layer reticulated domes, based on nonlinear equilibrium equations. Kato et al. [32] performed an analysis on buckling and developed an analytical method for steel reticulated domes with semi-rigid ball joints, on the basis of a nonlinear elastic–plastic hinge analysis equation for three-dimensional beam-columns with elastic–plastic hinges perfectly located at both ends and at the mid-span for each member.

However, while those studies focused on the response of the space structure under static load, little research has been carried out on the collapse mechanism of a space structure under seismic load. Under seismic load, damage accumulation induced by cyclic loading is a very important factor that needs to be considered for its effect on the numerical simulation of the deterioration of the stiffness of the dome, the strength of materials, and the fracture of members. In other words, an accurate prediction of the dynamical response of structures require serious attention to be paid to damage accumulation. Some researchers carried out similar studies on Glass Fiber Reinforced Polymer (GFRP) composites. Allah et al. [33] studied the effect of mean stress on the behavior of the S-N diagram of GFRP composites through constant deflection flexural fatigue tests on standard unidirectional glass fiber-reinforced polyester pultruded rods. Wang et al. [34] carried out an experimental study on the high-cycle (500,000 times) fatigue performance of a new type of GFRP composite material cross arm. Hyeong, et al. [35] investigated the degradation of GFRP composites using an accelerated aging method in which two types of E-glass/vinyl ester rods were exposed to moisture, chloride, alkali, and freeze–thaw cycling conditions for up to 132 days and found that the tensile properties of the GFRP rods were significantly reduced due to the degradation of GFR.

However, in terms of the structure of buildings, in most of the numerical models, damage accumulation is ignored, and little work has been done in this area.

In this paper, based on thermodynamic theory, the damage evolution equations for fiber beam elements derived by the authors [36] are used to simulate the progressive collapse mechanism of a single-layer cylindrical latticed shell. Both a corresponding constitutive relationship for beam elements and a relevant numerical analysis method are also developed. 

Due to the redundancy and indeterminacy of single-layer space structures, a dynamic instability criterion based on an implicit algorithm can capture neither the collapse mechanism of the reticulated shell nor the failure mode of individual components. Therefore, the simulation of the whole collapse process requires the application of an explicit dynamic algorithm, which is used in this paper. Based on the above studies, in the paper by Zhou et al. [36], the authors developed a subroutine program based on an explicit dynamic algorithm to analyze the response of the single-layer reticulated shell under a severe earthquake. The program was validated against experimental tests. It was proven that the proposed numerical method can accurately simulate the members’ failure, the redistribution of internal forces, and the collapse mechanism of the whole structure. Based on this numerical method, parametric studies on single-layer reticulated shells are performed and the collapse mechanism of this type of structure is studied. 

## 2. The Numerical Model

**Figure 1:**
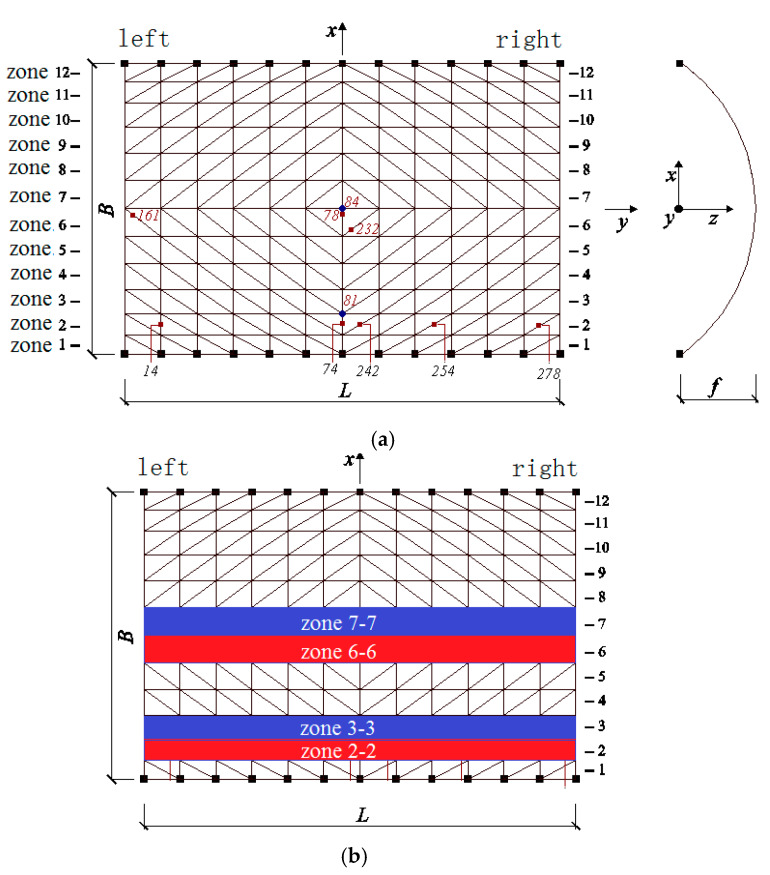
Single-layer cylindrical latticed shell with structural zone denoted: (**a**) Structural zone in the model; (**b**) Structural zone in the dome.

The collapse analysis is performed by the general-purpose program ABAQUS 6.12 by Dassault SIMULIA, through the further development of the subroutine VUMAT program in Abaqus. The numerical method introduced in Zhou et al. [36] is used in this research. All the structural members of the reticulated shell are simulated using the beam elements. Each beam element is further discretized into eight longitudinal fibers across their cross-section, with an appropriate constitutive model defined for each fiber, as shown in Figure 1.

In the dynamic analysis, the below damage criterion is used:

(1) $$dD=\frac{1-D}{2\sigma_{11}}d\sigma_{11}$$

where $\sigma_{11}$ is normal stress existing in a beam element, *D* is the cumulative damage.

The failure criterion of each fiber is determined based on [36]. When $D_{new}$ in a fiber develops into a certain value *D*_limt_, the fiber is determined to have failed. *D*_limt_ is determined using the below Equation:

(2) $$D_{new}=1-{\left(\frac{f_{u}}{f_{y}}\right)}^{-\frac{1}{2}}$$

where $f_{u}$ is the ultimate tensile strength, $f_{y}$ is the yield stress.

Based on the strain equivalence hypothesis, the following constitutive model was used in the simulation. In the elastic loading or unloading stage:

(3) $$\tilde{\varepsilon^{e}}=\tilde{\sigma }/E$$

In the plastic loading stage, the strain equivalence hypothesis is still valid and it is

(4) $$\varepsilon =\tilde{\sigma }/E_{t}=\sigma /{\tilde{E}}_{t}$$

where $\tilde{\sigma }=\sigma /\left(1-D\right)$ is the effective Cauchy stress tensor and $\tilde{E}=E\times (1-D)$ is the effective elastic tensor.

In the designed VUMAT subroutine program, when the failure of one fiber is triggered, the elastic modulus of this fiber will be set as zero. When all the fibers in a beam element fail, that beam element is judged as having failed; thus, this beam element will be deleted in the program. Therefore, the process of the collapse of the dome can be simulated. After an element is deleted due to failure, the explicit dynamic analysis based on the central difference method is performed, which enables the accurate capture of the response of the dome. For a detailed explanation of the numerical algorithm, please refer to Zhou et al. [36].

## 3. Progressive Collapse Analysis of Single-Layer Cylindrical Latticed Shell

Based on the aforementioned analysis method, a single-layer monoclinic cylindrical reticulated dome with a span of 20 m, 30 m in length and 7.5 m in height was modeled using the general-purpose program Abaqus (Figure 1). The dome was supported with pins along the two longitudinal edges, with bottom nodes hinged to the ground. All the structural members are Chinese steel tubular sections, where, the lateral and diagonal members are Chinese Ø166 × 5, and the longitudinal member are Chinese Ø114 × 5. The grade of the steel is Chinese Q235-B), with an initial yield stress of 235 MPa. Von Mises theory is adopted as yield criteria. The Young’s modulus was 2.06 × 10^5^ MPa. The Poisson ratio was 0.3. The live load of the roof was taken as 2.5 KN/m^2^. Figure 1 also shows the structural zone divided by the authors, which is shown to clearly demonstrate the response of the dome under earthquake loading. 

**Figure 2:**
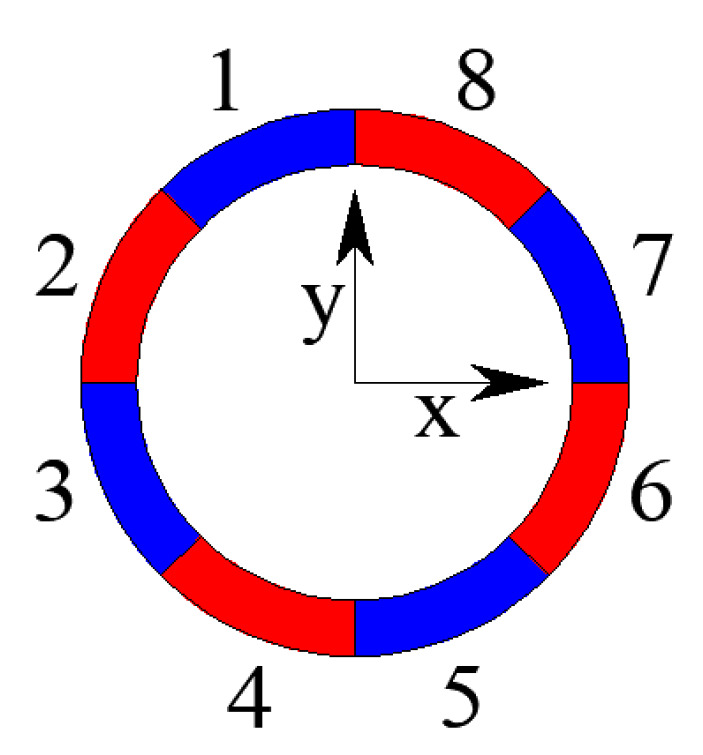
The location and numbering of the fibers in a single beam element.

In the simulation, all the structural members were modeled using beam elements. Each beam element was further divided into four segments along its length to model the buckling behavior. As is explained Zhou et al. [36], this is an effective way to model the member buckling with sufficient accuracy. In addition, each beam element was subdivided into eight fiber beams across its cross-section, as shown in Figure 2. The fibers are numbered from one to eight along the circumference of the beam element (Figure 2). 

**Figure 3:**
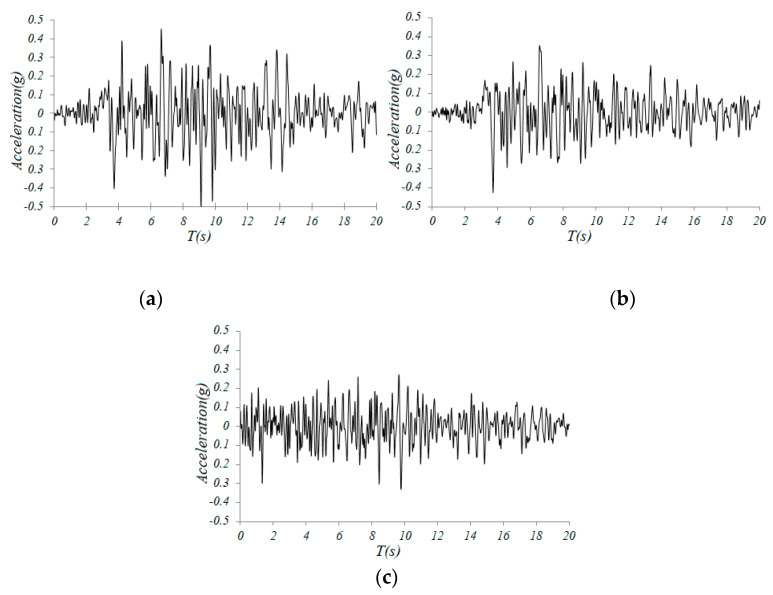
Amplified Taft earthquake wave: (**a**) X direction, (**b**) Y direction, (**c**) Z direction.

The analysis was divided into two steps. The first step was static analysis, where the gravity load was applied to the dome. The second step was dynamic analysis, where the time history of the Taft earthquake (Figure 3) was applied at the support, in X, Y, and Z directions, with the peak ground acceleration (PGA) of 0.5 g in the X direction, 0.85 × 0.5 g in the Y direction, and 0.65 × 0.5 g in the Z direction, respectively According to the Chinese earthquake design code [37], the scale factors of 3.211304, 2.370329 and 3.161574 were applied to the original time history in three directions, respectively. The time duration was 20 s, which is greater than 10 times the natural period of the dome. The time step was 0.02 s. The constitutive model of the material uses the hybrid hardening model and the modulus considered the cumulative effect of damage to simulate the performance deterioration of the material, which occurred during plastic tension in the hysteresis process under seismic loading.

**Figure 4:**
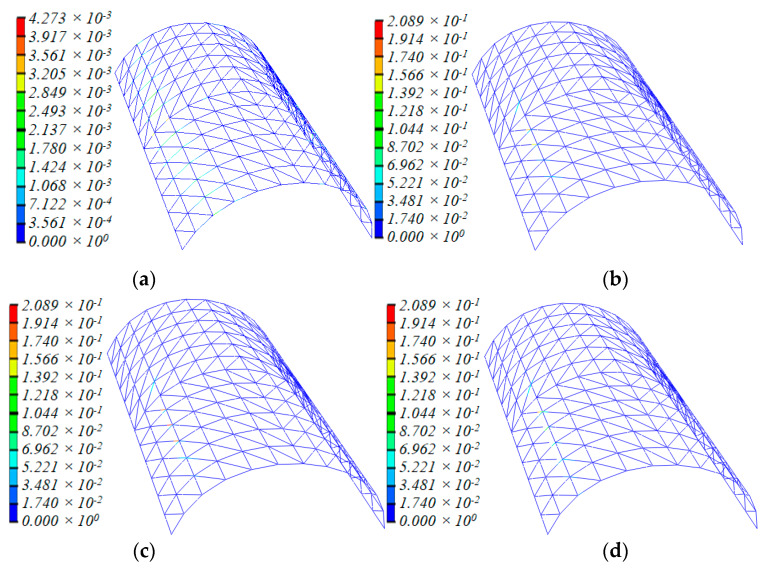

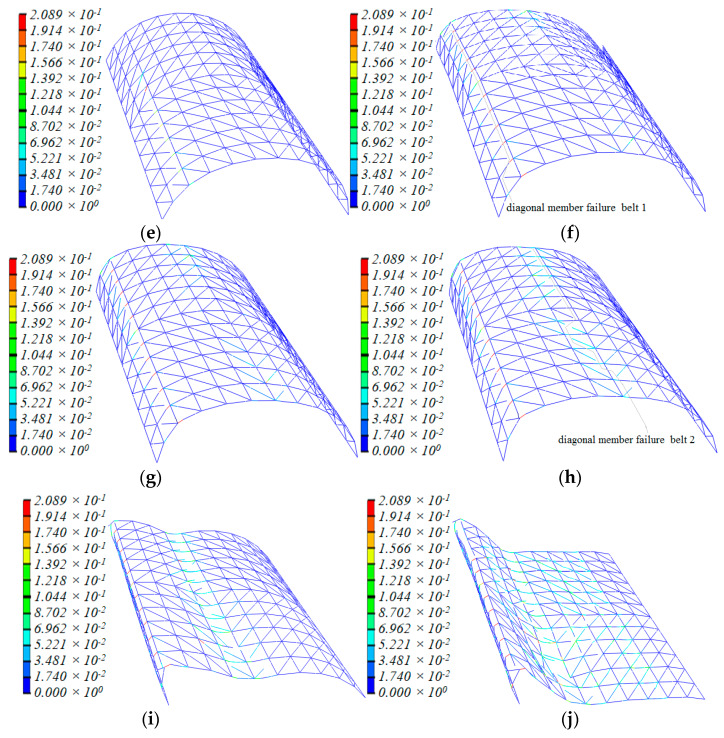
Deformation and D value distribution of structural members during the collapse process: (**a**) t = 4.56 s, (**b**) t = 6.16 s, (**c**) t = 11.78 s, (**d**) t = 11.80 s, (**e**) t = 12.03 s, (**f**) t = 14.38 s, (**g**) t = 15.40 s, (**h**) t = 15.83 s, (**i**) t = 16.38 s, (**j**) t = 16.78 s.

Figure 4 depicts the deformation and damage distribution during the collapse process, where the distribution of the *D* value can be checked. It demonstrates the process where the *D* value was zero, which indicates that no damage occurred until the rupture of the member when *D* > *D*_limt_. 

According to the analysis results, the dome entered the plastic stage at 1.88 s for the first time. Damage distribution spread to zones 2-2 and 3-3 and the symmetric zone of 6-6/7-7, but the *D* value was extremely small in this stage. With the ground motion continued, at 6.16 s, the *D* value of many diagonal and lateral members in zones 2-2 and 3-3, at adjacent ends, had obviously exceeded that of members in other zones (Figure 4b). By 11.78 s, the damage state of the whole dome had furtherly deteriorated. The *D* value of damaged structural members kept rising and more diagonal and lateral members were damaged. At 11.80 s, three diagonal bars in zone 2-2 failed at the ends of the joints with a threshold value of 0.209.

At 12.03 s, all the diagonal members in zone 2-2 failed, while the damage range spread to the left of the zone, with the damage area increased dramatically, although no member facture was observed. At 14.38 s, all the diagonal members in zone 2-2 failed because of continuous damage accumulation, and the failed zone was like a continuous and regular “diagonal member failure belt”. Lateral bars were left unbroken and the damage area continue to propagate. The deflection of the dome had been significant. Furthermore, some lateral members and diagonal members in zone 6-6/7-7 were damaged.

Afterwards, the damage area was found between zones 6-6 and 7-7. At 15.40 s, most diagonal members in the zone were damaged and a few at two longitudinal ends failed. By 15.83 s, most diagonal members in zone 6-6 and two diagonal members at the right of zone 7-7 had failed. The position of structural members failed in a similar manner to the “diagonal member failure belt “with its width becoming slightly wider than when it first came into being (Figure 4h). No lateral members failed, though they were obviously damaged. Furthermore, the overall downward deflection of the dome became significant during this process. With the continuous action of the earthquake, the dome began to collapse downward rapidly. Horizontally, the dome collapsed towards the position of the first “diagonal member failure belt”, the whole dome now looked like an italic letter “M”. Finally, 1.13 s later, with the formation of the second “diagonal member failure belt”, the dome collapsed totally. The D value of longitudinal bars of the dome had not increased much throughout.

### 3.1. Collapse Pattern of “Double-Diagonal Member Failure Belt”

**Figure 5:**
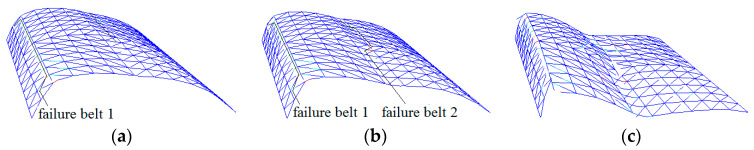
Collapse process of cylindrical reticulated domes of different span/depth ratios at typical times (span/depth = 4:1): (**a**) belt 1 formed, (**b**) belt 2 formed, (**c**) dome collapses.

**Figure 6:**
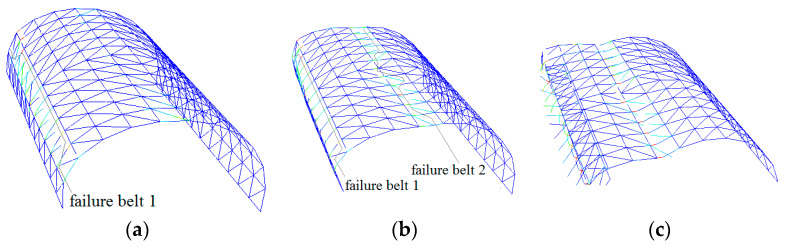
Collapse process of cylindrical reticulated domes of different span/depth ratios at typical times (span/depth = 2:1): (**a**) belt 1 formed, (**b**) belt 2 formed, (**c**) dome collapses.

**Figure 7:**
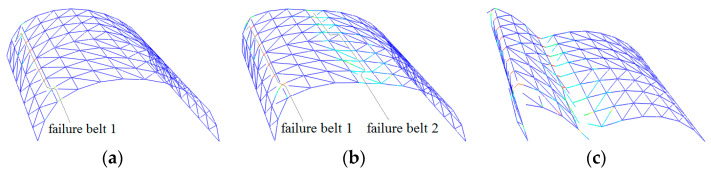
Collapse process of cylindrical reticulated domes of different length/width ratios at typical times (length/width = 1:1): (**a**) belt 1 formed, (**b**) belt 2 formed, (**c**) dome collapses.

**Figure 8:**
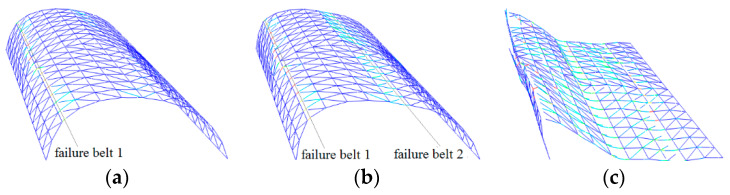
Collapse process of cylindrical reticulated domes of different length/width ratios at typical times (length/width = 2:1): (**a**) belt 1 formed, (**b**) belt 2 formed, (**c**) dome collapses.

Figure 5 and Figure 6 depict the deformation and distribution of the damage of the structural member during the collapse process for the other two domes, with span:depth ratios of 4:1 and 2:1, respectively. Their spans are kept at 20 m. Figure 7 and Figure 8, respectively, depict the other two 20-m-span domes, one with a length:width ratio of 1:1, another with a ratio of 2:1. With the increase in the length:width ratio, the position of the “diagonal member failure belt” moved from zones 2-2/6-6 to 3-3/7-7. The decrease in the length:width ratio caused a contrasting trend. With the exception of the above, the basic process of collapse and final collapse forms are similar. 

**Figure 9:**
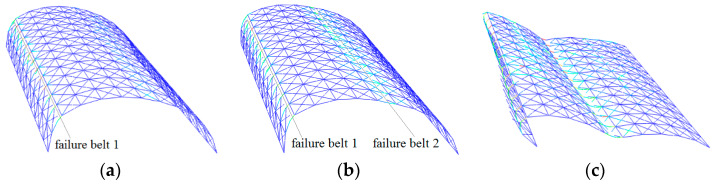
Double diagonal member failure belt: (**a**) belt 1 formed, (**b**) belt 2 formed, (**c**) dome collapses.

Figure 9 depicts the failure mode of double-diagonal member cylindrical reticulated domes, which are similar to single-diagonal member cylindrical reticulated domes.

Apart from the above analysis, extensive parametric studies were performed by the authors with different spans, different span:depth ratios, different length:width ratios and different roof loadings. We have proven that similar collapse processes were observed.

In addition to the above case studies, all the analysis cases were applied with a peak ground acceleration (PGA) of 0.5 g in the Y direction, 0.85 × 0.5 g in the X direction, which is different to the first loading regime. The results showed that nothing, apart from the time when the dome collapsed, changed.

**Figure 10:**
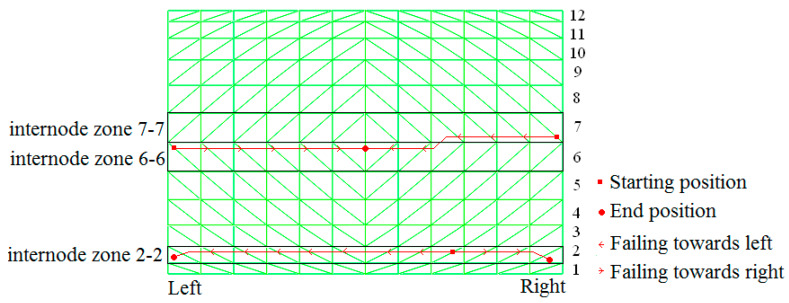
The demonstration of the collapse mechanism of the shell.

Therefore, a consistent failure pattern for the collapse process of single-layer cylindrical reticulated domes can be concluded. Under severe earthquakes, serious damaged first occurs at 1/4 height of the dome. Diagonal members failed continuously from the center to the boundary lengthwise and the first “diagonal member failure belt” came into being (Figure 10). The stiffness of the dome also greatly weakened. Then, another damaged zone appeared at the middle of the dome and, in this zone, diagonal members failed continuously lengthwise from the ends to the center. A second “diagonal member failure belt” was formed. The stiffness of the dome was reduced further. The whole deflection became significant. The position of the “belt” varied slightly with the change in geometry. In the final stage, the dome deflected rapidly at the second “belt”, dropping towards the first “belt”. The dome collapsed. We call this collapse process and phenomenon the “double-diagonal -members-failure-belt” pattern.

### 3.2. Mechanism of Progressive Collapse Process

Earthquake loading causes deformations and material damage. Deformation and damage cause a variety of mechanical characteristics of dome. The variety deeply affect the deformation and development of material damage in turn (“failure” is the extreme state). This kind of complex relationship exists and works during the whole process.

According to the analysis results in the earlier section, it can be concluded that, of the deformation and mechanical characteristics of dome changed along the whole process, different of deformation characteristics and mechanism properties were observed, the collapse process can be divided into three stages. 

**Stage 1**: This stage refers to the process from the earthquake occurring to the formation of the first “belt” (0–14.38 s). During this stage, the phenomenon of material damage and diagonal members failing mainly occur in zone 2-2.

The cylindrical reticulated dome, with longitudinal edges supported with pins, is one type of space structure that exhibits planary arch features. The response of dome under severe earthquake is very similar to that of a planary arch. The diagonal members are for bracing, and the longitudinal member works as a tie bar, which coordinates the continuity of the vertical and horizontal deformation. Therefore, during earthquakes, the axial force, bending moment and torsion of each type of bar vary with time, although the overall characteristics are relatively stable.

Thus, at the beginning of an earthquake, the maximum internal force can be observed at the 1/4 span of the dome from the boundary, and the member that first developed into a plastic state and caused the material damage must come from this zone. 

**Figure 11:**
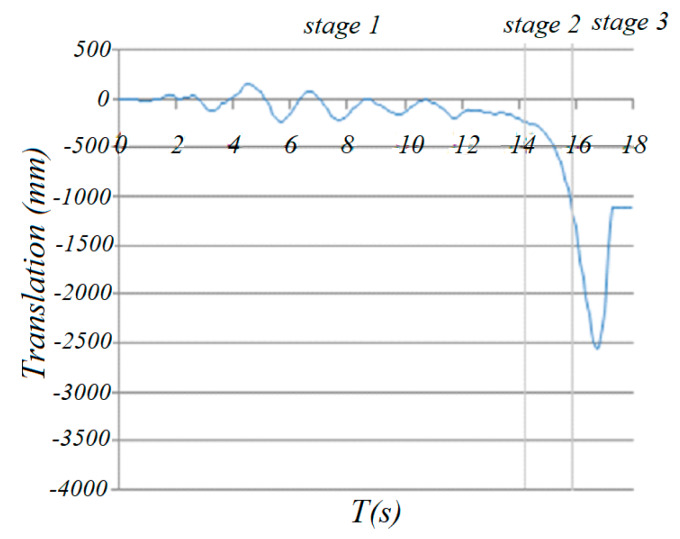
The horizontal translation of gravity center in X direction.

However, Earthquake loading is asymmetric, so plastic deformation and damage accumulation in the dome are also inevitably asymmetric. This means the *D* value and the increasing speed of the *D* value of the bars in this zone are larger than those of bars in other zones. With the continuing ground motion and gradually increasing amplitude, 5.13 s later, the center of gravity of the dome moved towards the X direction (Figure 11). This is because plastic deformation occurred due to vibrations, and the overall geometric configuration shifted towards zone 2-2. This phenomenon conversely caused the internal forces of members in zone 2-2/3-3 to become significantly larger than those in the symmetric zone of 11-11/10-10. Consequently, the speed of damage of bars in zone 2-2 was higher than that of bars in the symmetric zone (Figure 4b,c).

**Figure 12:**
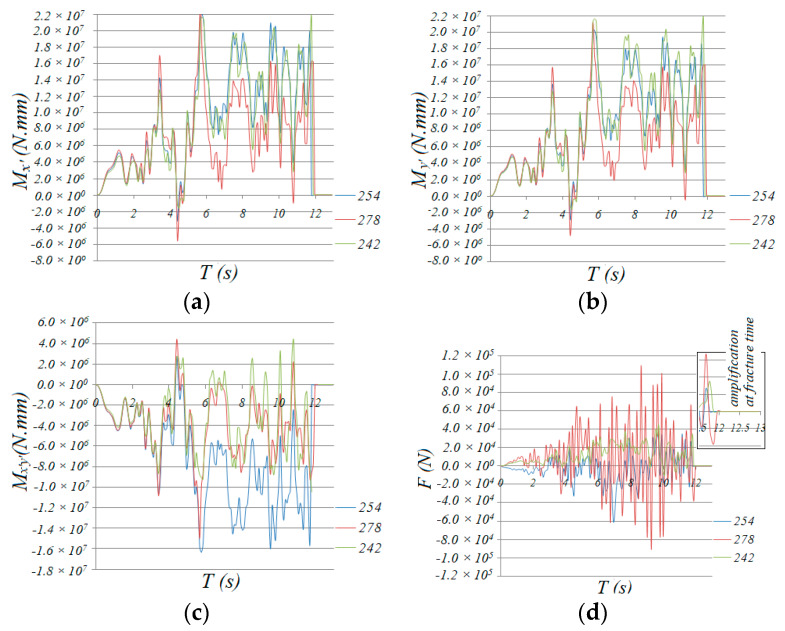
Time history of the internal force of the diagonal members: (**a**) *M*_x’_, (**b**) *M*_y’_, (**c**) *M*_x’y’_, (**d**) *F*.

As shown in Figure 12, through a comparison of the time history curves of bending moments *M*_x’_ and *M*_y’_, torsion *M*_x’y’_ and axial force *F* of members 242/254/278 in zone 2-2, it can be found that, although the axial force *F* of 254 was less than that of 242, the bending moments *M*_x’_ and *M*_y_ and torsion *M*_x’y’_ of 254 are obviously larger than those of 242. The axial force and bending moments of 278 are approximate to those of 254, but the torsion of 278 is less than that of 242. So, the D values of the slant bar of 254 and neighboring bars in zone 2-2 are obviously larger than those of other bars (Figure 4b,c).

**Figure 13:**
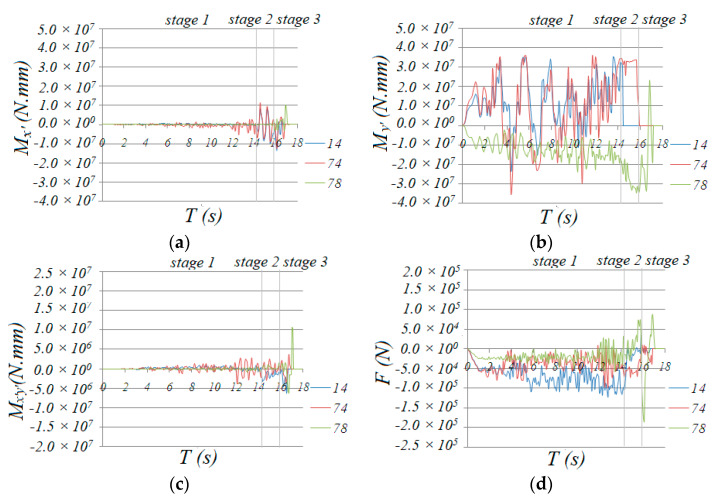
Time history of internal forces of traverse bar 14, 78 and 74: (**a**) *M*_x’_, (**b**) *M*_y’_, (**c**) *M*_x’y’_, (**d**) *F*.

**Figure 14:**
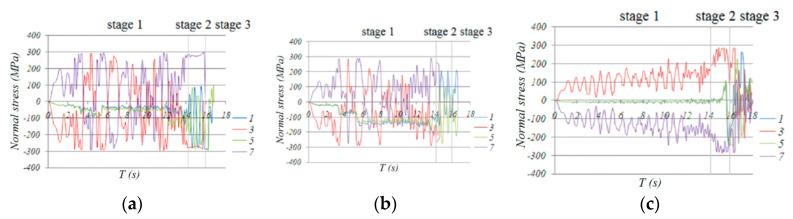
Time history of normal stress in fiber 1,3,5,7 of traverse bar 14, 78 and 74: (**a**) bar 74, (**b**) bar 14, (**c**) bar 78.

According to Figure 13, which shows the variety of internal forces of traverse bars 14, 78 and 74 at the center of zone 2-2, the traverse bars are mainly subjected to bending moment *M*_y_, while axial force F, bending moment *M*_x’_ and torsion *M*_x’y’_ at this stage are minimal, so *M*_y’_ contributed mainly to the damage increment. This is why, in Figure 14, the normal stresses of upper section fiber 3 and lower section fiber 7 were much larger than those of middle section fibers 1 and 5, which hardly exceeded the yield point and caused corresponding material damage. Thus, damage development concentrated in fiber 3 and 7 and traverse bars hardly failed. This is the common response observed in all the transverse bars of the monoclinic and double-skew cylindrical reticulated domes under the same ground motion.

**Figure 15:**
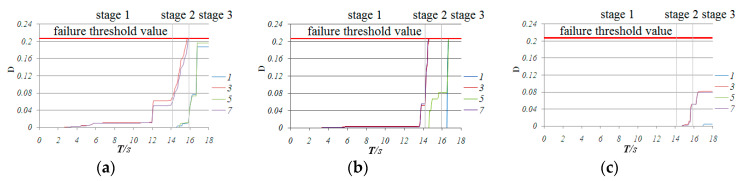
Time history of D value in fiber 1,3,5,7 of bar 14, 78 and 74: (**a**) bar 74, (**b**) bar 14, (**c**) bar 78.

Based on the above analysis, bar 254 and neighboring bars failed at first. Furthermore, once they lost their bearing capacity, the internal force redistribution effect imposed a force on the neighboring bars that had not yet failed (as shown in Figure 14a), and the D value developed rapidly until the bar failed (Figure 15). Then, the internal force redistribution effect worked again. With the failure propagating, the force redistribution to the neighboring bars continued. Finally, all the bars in zone 2-2 failed. The collapse process then entered the second stage.

**Figure 16:**
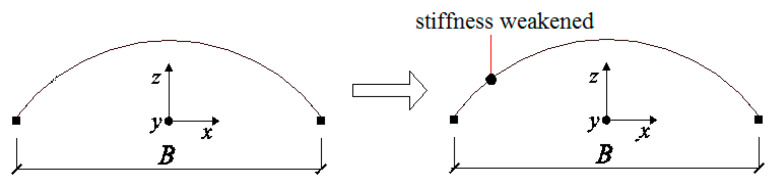
The transition between stages 1 and 2.

**Stage 2:** This refers to the stage from the formation of the first “belt” in zone 2-2 to the formation of the second “slant-bars-failed-belt” in zone 6-6/7-7 (14.38–15.83 s). In this stage, the phenomena of material damage accumulation and the failure of the bars mainly occurred in zones 6-6 and 7-7. The effect of material damage accumulation caused not only the failure of all diagonal members in zone 2-2, but also the significant deterioration of flexural stiffness in fibers 3 and 7 in the traverse bars. At this time, the dome acted as an arch whose local flexural stiffness in zone 2-2 weakened significantly (Figure 16).

**Figure 17:**
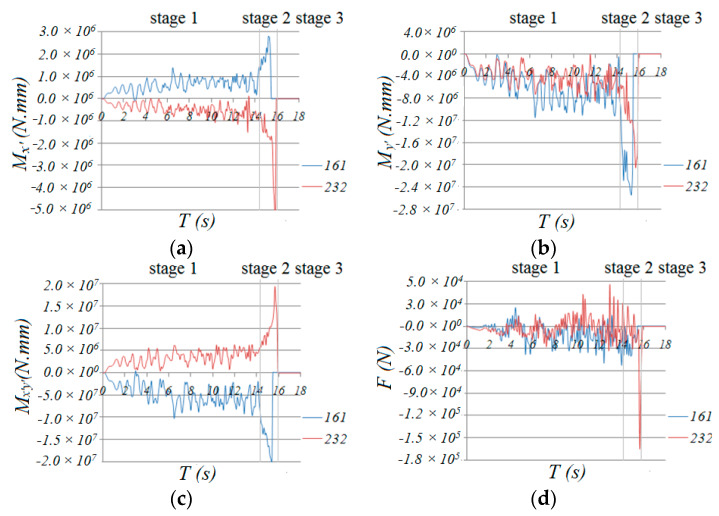
Time history of internal forces of diagonal bar 161and 232: (**a**) *M*_x’_, (**b**) *M*_y’_, (**c**) *M*_x’y’_, (**d**) *F*.

In Figure 14a, we can see that the normal stress at upper fiber 3 and lower fiber 7 at the end section of traverse bar 74 was maintained at a value of 280 MPa, which was much greater than the initial yield stress of 235 MPa, while the normal stress of fibers 5 and 1 also increased dramatically, acting as a plastic hinge. Fiber 3 and 7 of traverse bar 14 failed at a short time after entering this stage, but the stress of fibers 1 and 5, whose stress was relatively small, increased rapidly to around 200 MPa. We can conclude that the normal stresses of the remaining fibers of bar 14 were larger than the yield point. This means that the end of bar 14 acted as a plastic hinge as well. Finally, we can conclude that all the traverse bars in zone 2-2 that had not yet failed acted as plastic hinges. The formation of plastic hinges induced significant internal force redistribution effect. As shown in Figure 17, the bending moment *M*_y’_ axial force F, bending moment *M*_x’_ and torsion *M*_x’y’_ of diagonal bars 161 and 232 (Figure 1), one at the end and the other at the mid of zone 6-6, were less than those of bars 242, 254 or 278 (Figure 12) in stage 1. Thus, almost no damage was done to these bars. However, the value of *M*_y’_, *M*_x’_, torsion *M*_x’y_, began rapid increasing since the formation of the first “belt”.

Therefore, continuously increasing the internal force induced by ground motion resulted in severe damage to the diagonal bars 0.8 s later when entering the second stage, because the internal forces of the bars at two ends were larger than that of the bars in the middle; the bars at the ends of the nodes of zone 6-6 failed first (Figure 4g). Then, the other bars failed successively from both ends to the middle in a short time. Finally, at 15.83 s, the second belt was formed.

For traverse bar 78 in zone 6-6, although the internal forces of the bar significantly increased because of force redistribution, the damage accumulation mainly developed in upper fiber 3 and lower fiber 7, while fibers 1 and 5 were not damaged seriously. Thus, no bars failed in all fibers. In Figure 14c, the normal stresses in fibers 3 and 7 in bar 78 were very high; we could conclude that this bar acted as a plastic hinge as well.

The successive failure of diagonal members and damage to traverse bars in zone 6-6 greatly weakened the vertical stiffness of the dome. Thus, under the earthquake loading, the dome deflected downwards significantly. At the same time, angular displacement was observed in the traverse bars in zone 2-2, which made damage in fibers 3 and 7 develop rapidly (Figure 14).

**Figure 18:**
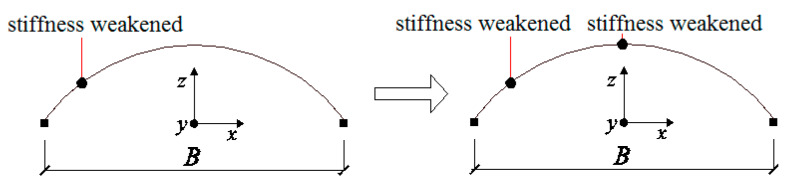
The transition between stages 2 and 3.

Up to now, the generation of the second “diagonal member failure belt” and the existence of the first “diagonal member failure belt” caused the formation of two zones whose stiffnesses were seriously weakened, or two plastic hinges. As shown in Figure 18, the lateral mechanism characteristics of the dome changed again.

**Figure 19:**
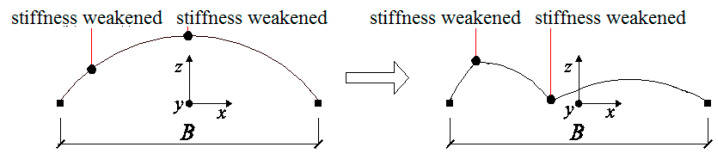
The collapse of the dome in stage 3.

**Stage 3**: This refers to the process of transitioning from the formation of the second “diagonal member failure -bar-failed belt” to the final collapse of the dome (15.83 s~ end). This process is mainly an irreversible collapse process of the whole geometric configuration of the dome. In “belt” two, the loading is borne and transferred only by the seriously damaged traverse bars. With the development of damage and the deterioration of angle stiffness of traverse bars in the vertical plane, the dome acted as an almost unstable system. Then, under an earthquake, total collapse occurred, as shown in Figure 19. The calculation results showed that the process is very rapid and irreversible.

Traverse bar 14 and the neighboring bars in zone 2-2 rapidly failed because of suddenly increasing torsion. Then, the bending moment *M_y’_* of bar 74 obviously increased and made the damage value of fibers 1 and 5 increase.

In conclusion, in the three stages, the variation in the mechanical characteristics, damage zone and deformation status of the dome show distinct differences. However, we should understand that the three stages are closely related rather strictly divided.

### 3.3. Displacement during the Collapse Process

**Figure 20:**
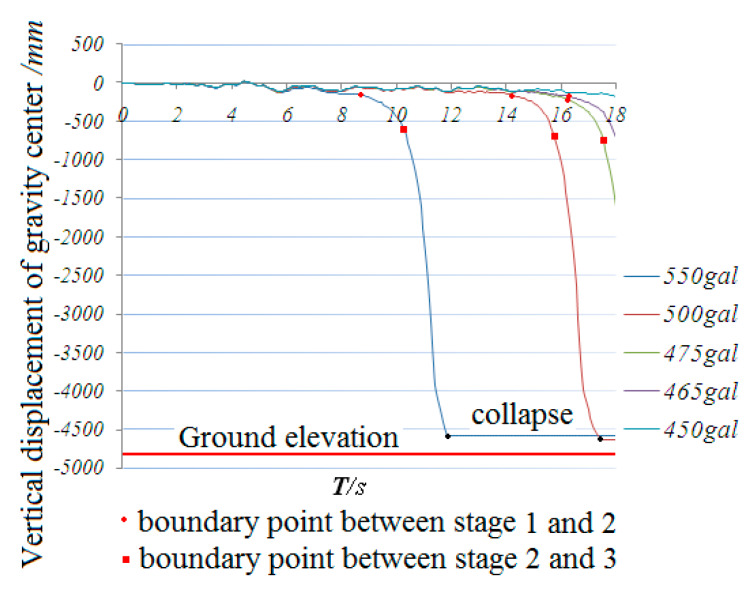
Time history of vertical translation of gravity center.

The deformation of the dome changed throughout the whole collapse process; therefore, the center of the structure changed as well. Figure 20 shows the change in the locations of the center of gravity of the structure during the whole collapse process. Different peak ground acceleration values were selected with the same duration of 18 s. It can be seen that the center of gravity decreased with the different PGA values.

When PGA was 0.45 g, the damage was observed at 1.9 s and the rupture of the first diagonal bar occurred at 16.44 s. However, the failure belt was not formed until 18 s. At 1.9 s, the location of the center of the gravity fluctuated and no significant acceleration in the center of gravity was observed.

When PGA was 0.465 g, the first diagonal member failure belt formed at 16.55 s. The second belt started to form at 17.63 s. However, the failure belt was not formed until 18 s. It can also be observed that the slope of the curve in Figure 17 increased dramatically. 

When PGA was 0.475 g, the rupture of the first diagonal member failure belt formed at 16.38 s. The second belt started to form at 17.48 s. However, it can also be observed that the slope of the curve increased at stage 2 compared to stage 3. When PGA was 0.5 g, the rupture of the first diagonal member failure belt occurred at 14.38 s. The second belt started to form at 15.83 s.

It can also be seen that when PGA was 0.475 g, the acceleration distance of the center reached 1286mm; however, for a larger PGA of 0.5 g, when the time was shorter than 16 s, the acceleration distance of the center was smaller than 1286 mm, which indicates the effect of the duration on the time history.

Based on the above analysis, the following can be concluded:

1. At the first stage, the descent of the center of gravity is slow;
2. The descent of the center of gravity speeds up from stage 1 to stage 3;
3. The time to reach stage 2 or 3 is determined by the peak ground acceleration applied to the structure—the larger the PGA, the quicker it will reach stage 2 or 3;
4. The time duration will determine the collapse mechanism of the dome, which is one of the dominant factors.

## 4. New Enhanced Schemes to Mitigate the Collapse 

**Figure 21:**
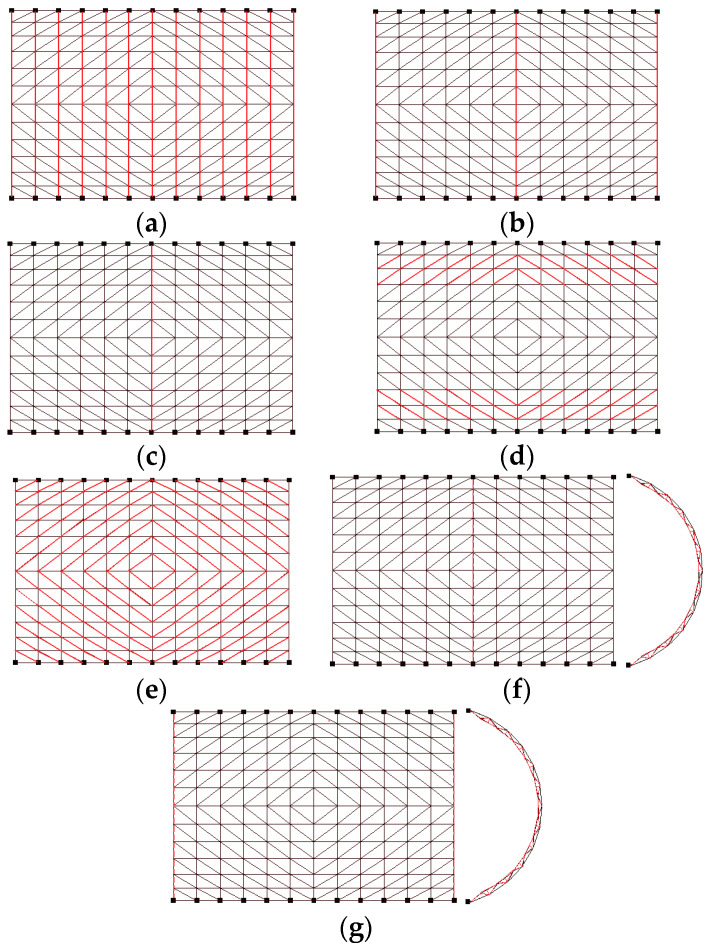
Seven different schemes to enhance the dome: (**a**) scheme 1, (**b**)scheme 2, (**c**) scheme 4, (**d**) scheme 4, (**e**) scheme 5, (**f**) scheme 6, (**g**) scheme 7.

Based on the numerical simulation results, seven mitigation schemes were developed to prevent the collapse of the dome: in Figure 21a, the cross section of all top chords in the transverse direction increased. In Figure 21b, the cross section of three top chords in the transverse direction increased. In Figure 21c, the cross section of the top chords at center was increased. In Figure 21d, the cross section of the diagonal members as shown in the Figure increased. In Figure 21e, the cross section of all the diagonal members increased. In Figure 21f, extra diagonal and bottom cords were added to the arch at the center of the shell. In Figure 21g, extra diagonal and bottom cords were added to the two arches at the end of the shell. All the increased section sizes used the ones specified in GBT17395-1998 [38], with a wall thickness of 6 mm.

**Note:** Red solid line represents the enhanced bars; the red dotted line indicates the plane arch truss. The height of the truss section is 450mm, and the diameter of the truss web members is Φ30 × 3.5 

**Figure 22:**
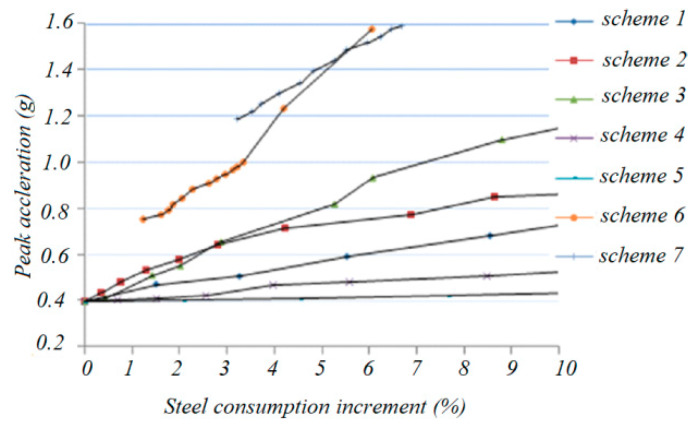
Relation between collapse peak acceleration vs. steel consumption increment.

**Figure 23:**
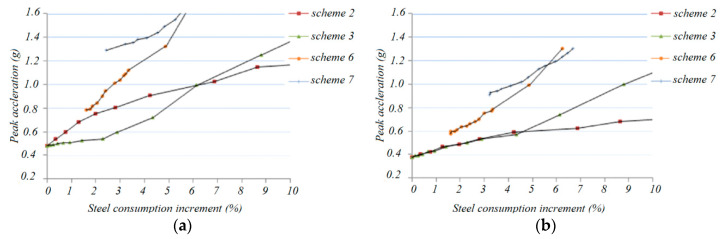
Curve of acceleration vs. steel consumption increment under various live loads: (**a**) proof live load 1.3 KN/m^2^, (**b**) proof live load 1.7 KN/m2.

A time history analysis was performed for all seven enhanced schemes. The results are shown in Figure 22 and Figure 23. Figure 22 shows the relationship between the various earthquake inputs with the increase in the use of the steel material.

It can be seen that, with the increase in the steel consumption, the collapse peak acceleration, which can be taken as the seismic capacity index of schemes 6 and 7, increases most rapidly. On the other hand, for schemes 2 and 3, the capacity index increases less than in schemes 6 and 7, while in schemes 4 and 5, the capacity index increases the least.

Figure 23 shows the relationship between the various live loads (1.3 KN/m^2^ and 1.7 KN/m^2^) on the roof with the use of the steel material after the analysis. It can be seen that scheme 7 shows a reasonably high earthquake resistance with a small increase in steel usage.

## 5. Conclusions

In this paper, a detailed progressive collapse analysis of a single-layer cylindrical latticed shells under severe earthquakes was performed using the fiber beam element method with the inclusion of the accumulation of material damage. The below conclusions can be made:

1. A failure pattern called “double-diagonal -members-failure-belt” is discovered for this type of space structure;
2. The failure pattern can be divided into three consecutive stages:
  - Significant variation in the deformation in different structural zones;
  - Continuously increasing internal forces;
  - Damage to structural members.
